# Construction and implications of structural equation modeling network for pediatric cataract: a data mining research of rare diseases

**DOI:** 10.1186/s12886-017-0468-5

**Published:** 2017-05-19

**Authors:** Erping Long, Shuangjuan Xu, Zhenzhen Liu, Xiaohang Wu, Xiayin Zhang, Jinghui Wang, Wangting Li, Runzhong Liu, Zicong Chen, Kexin Chen, Tongyong Yu, Dongxuan Wu, Xutu Zhao, Jingjing Chen, Zhuoling Lin, Qianzhong Cao, Duoru Lin, Xiaoyan Li, Jingheng Cai, Haotian Lin

**Affiliations:** 1 State Key Laboratory of Ophthalmology, Zhongshan Ophthalmic Center, Sun Yat-sen University, Guangzhou, 510060 China; 2 School of Mathematics, Sun Yat-sen University, Guangzhou, 510275 China; 3 School of Public health, Sun Yat-sen University, Guangzhou, 510080 China

**Keywords:** Rare diseases, Data mining, Pediatric cataract, Structural equation modeling

## Abstract

**Background:**

The majority of rare diseases are complex diseases caused by a combination of multiple morbigenous factors. However, uncovering the complex etiology and pathogenesis of rare diseases is difficult due to limited clinical resources and conventional statistical methods. This study aims to investigate the interrelationship and the effectiveness of potential factors of pediatric cataract, for the exploration of data mining strategy in the scenarios of rare diseases.

**Methods:**

We established a pilot rare disease specialized care center to systematically record all information and the entire treatment process of pediatric cataract patients. These clinical records contain the medical history, multiple structural indices, and comprehensive functional metrics. A two-layer structural equation model network was applied, and eight potential factors were filtered and included in the final modeling.

**Results:**

Four risk factors (area, density, location, and abnormal pregnancy experience) and four beneficial factors (axis length, uncorrected visual acuity, intraocular pressure, and age at diagnosis) were identified. Quantifiable results suggested that abnormal pregnancy history may be the principle risk factor among medical history for pediatric cataracts. Moreover, axis length, density, uncorrected visual acuity and age at diagnosis served as the dominant factors and should be emphasized in regular clinical practice.

**Conclusions:**

This study proposes a generalized evidence-based pattern for rare and complex disease data mining, provides new insights and clinical implications on pediatric cataract, and promotes rare-disease research and prevention to benefit patients.

**Electronic supplementary material:**

The online version of this article (doi:10.1186/s12886-017-0468-5) contains supplementary material, which is available to authorized users.

## Background

Rare diseases are regarded as one of the main global disease burdens worldwide. Most of rare diseases are considered as complex diseases that are caused by a combination of multiple morbigenous factors [1]. However, uncovering the complex etiology and pathogenesis of rare diseases is difficult because of limited clinical resources and conventional statistical methods. Therefore, there is an urgent need to build a network combining multidimensional rare-disease data with innovative computational methods [2, 3].

Pediatric cataract is a typical rare disease with significant risk of visual loss [4]. Risk factors, systematic symptoms, and multi-dimension clinical evaluations are simultaneously indispensable for the pediatric cataract prevention and treatment process [5–9]. Therefore, pediatric cataract, which jointly combined the intricate pathogenesis and intractable clinical situation, is a suitable test case for the exploration of computational modeling and data mining for rare diseases.

Structural equation modeling (SEM) is a multivariate statistical technique that incorporates factors and path analysis [10], which is able to handle not only measurable variables, but also latent factors that cannot be measured or observed on their own. Latent variables, frequently encountered in substantive researches, are needed to be expressed by several measurable variables. However, most existing modeling techniques, such as multiple regression and observed variable analyses, cannot deal with latent variables whilst SEM compensates for these issues. Moreover, SEM can accurately measure unreliable events because it takes the measurement errors into account. Therefore, SEM has multiple advantages for the modeling of complex processes beyond simple correlations.

To explore the feasibility of applying SEM for the data mining of rare diseases, we established a pilot rare-disease specialized care center [Childhood Cataract Program of the Chinese Ministry of Health (CCPMOH)] to systematically record all basic information and the entire treatment process for pediatric cataract patients [11]. These clinical records contain the medical histories, multiple structural indices, and comprehensive functional metrics. We constructed a two-layer SEM network aiming to explore the interrelationship and the effectiveness of these potential factors and to provide clinical implications for pediatric cataract. We hope that this study will propose a generalized evidence-based pattern and valuable reference for rare and complex disease research.

## Methods

**Fig. 1:**
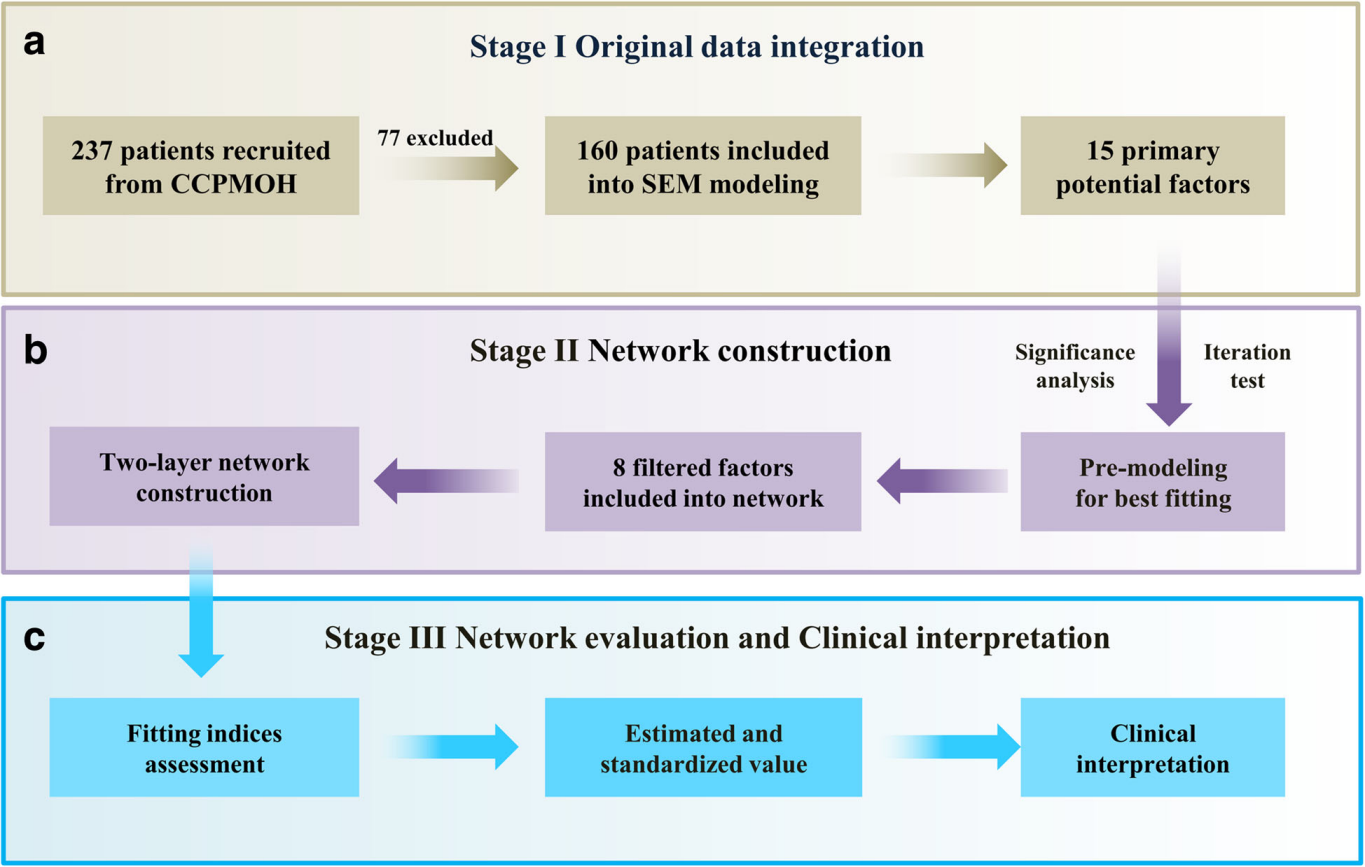
Pipeline of the study. The study pipeline consists of three sections. **a** for the first stage in original data integration, 15 potential factors of 160 CCPMOH patients were included into analysis (237 patients recruited with 77 excluded due to incomplete or missing clinical record). All the included patients are diagnosis with pediatric cataract. **b** for the second stage, after pre-modeling, 8 filter factors were included into the final two-layer network construction. **c**, for the third stage, fitting indices (χ^2^ et al.) were used for the network evaluation. Estimated and standardized values were obtained after evaluation. Finally, these values accompanied with previous clinical evidences could be translated into clinical interpretations. *CCPMOH* Childhood Cataract Program of the Chinese Ministry of Health

The methods portion of the pipeline consists of three sections. The first section provides the procedure for the original data integration, description, and definition. The second section includes a detailed methodology for SEM network construction. The third section is concentrated on the network evaluation indices and contribution of factors, which could serves as a reference for clinical interpretation. Each section is described below. The study pipeline is presented in Fig. 1.

### Study population

A total of 237 patients registered with the CCPMOH [11] were recruited from Zhongshan Ophthalmic Center [12], one of China’s largest eye hospitals, located in Guangzhou city, south China. Seventy-seven patients with wide range of incomplete or missing clinical record were excluded. All the included patients are diagnosed with pediatric cataract according to the International Statistical Classification of Diseases and Related Health Problems 10th Revision (ICD-10) Version (Disease numbers: H26.0 and Q12.0) [12]. Finally, 160 patients’ data records were included into the analysis and SEM construction. The dataset was anonymized throughout the research.

### Ethics approval

The research protocol was approved by the Institutional Review Board/Ethics Committee of Sun Yat-sen University (Guangzhou, China). Informed written consent was obtained from at least one family member of each participating patient, and the tenets of the Declaration of Helsinki were followed throughout this study. To allow confidential evaluation of the use of a slit-lamp, a Tono-Pen, a Pentacam imaging system and the Teller visual acuity (VA) cards in this study, this trial was registered with the Clinical Research Internal Management System of CCPMOH. The authors affirm that all ongoing trials and trials related to this study are registered.

### Examination protocol and variable definitions

All original variables and their SEM constructions are displayed in Table 1.

**Table 1:** Summary of original variables and SEM constructions

Two-layer Variables	One-layer Variables	Original Variables
Overall index	Concomitant variables	Age at diagnosis
		Laterality
		Height
		Weight
		Family heredity history
		Abnormal parturition history
		Abnormal pregnant history
	Structural indices	AL
		Area
		Density
		Location
	Functional indices	Ocular complications
		IOP
		UCVA
		BCVA

*AL* Axial length, *IOP* Intraocular pressure, *UCVA* uncorrected visual acuity, *BCVA* best-corrected visual acuity

The age at diagnosis, height, weight, family hereditary history, abnormal parturition history, and abnormal pregnancy history were collected in regular clinical practice. The diagnosis of pediatric cataract was made by experienced ophthalmologists according to the ICD-10 Version [13]. The family hereditary history was defined as any similar disease history of immediate family members. The abnormal parturition history included but was not limited to an abnormal fetal position, abnormal placenta and amniotic fluid, fetal distress, breech birth, fetal macrosomia, and hypamnion. The abnormal pregnancy history included premature delivery, post-term pregnancy, pregnancy complicated with infection, gestational hypertension and gestational diabetes mellitus.

Area, density, and location are three critical lesion indices that are defined for the comprehensive evaluation and treatment decisions of pediatric cataracts (details definition in Additional file 1) [14].

Laterality was classified as bilateral cataracts or a unilateral cataract. For unilateral cataracts, data from the affected eye were included in the analysis and modeling. For bilateral cataracts, if the interocular lesion (area, density, and location) presented no differences, only the variables of the right eye were chosen for the modeling and analysis; otherwise, data from this patient were excluded to avoid bias caused by reduplicative datasets.

The ocular complications consisted of microphthalmia, micro- or megalocornea, keratoconus, glaucoma, traumatic or complicated cataracts, or vitreous and retinal diseases. Otherwise, the data were regarded as “no ocular complications”.

The intraocular pressure (IOP) measurement was conducted using a Tono-pen tonometer (Reichert Inc., Seefeld, Germany) (details in Additional file 1) [15, 16].

#### Axial length (AL)

The contact A scan (B-SCAN-Vplus/BIOVISION, Quantel Medical, Clermont–Ferrand, France) was used to obtain the AL measurements (details in Additional file 1).

#### VA – Uncorrected visual acuity (UCVA) and best-corrected visual acuity (BCVA)

For preverbal children, a complete set of Teller VA Cards (Stereo Optical Company, Inc., IL, USA) was used to measure the monocular grating acuity of the eyes with cataracts [17]. The set consisted of 15 cards with gratings ranging in spatial frequencies from 0.32 cycles/cm to 38 cycles/cm in half-octave steps as well as a low vision card and a blank gray card. The infant was assessed using the standard procedure of the operation manual [18, 19]. For verbal children, visual functions were measured and recorded using a LEA Symbols 13-Line Translucent Distance Chart (Good-Lite Co., IL, USA) according to the standard procedure. The BCVA was assessed using spectacles after the UCVA examination. The results were translated into log MAR VA for analysis and modeling [20].

#### Quality control

Three experienced pediatric ophthalmologists (H.T.L., J.J.C. and Z.L.L.) performed all examinations according to our study protocols.

### Structural equation modeling

For the pre-modeling, the age at diagnosis, height, weight, family hereditary history, abnormal parturition history, and abnormal pregnancy history were considered the concomitant variables. The AL, area, density, and location were loaded into the one-layer latent variance and termed the structural index (STR). The ocular complications, IOP, UCVA and BCVA were loaded into another one-layer latent variance and termed the functional index (FUN). The overall index (OVE) was settled as the two-layer latent variance. The aforementioned structure for pre-modeling is presented in Table 1.

In the pre-modeling process, primary significance analysis and iteration testing were applied for variable fitting. The combination of the variables for the best SEM fitting will be accept and proceeded into further analysis. After pre-modeling, a total of 8 filtered variables were selected and included in the final SEM (Table 2).

**Table 2:** Summary of the distribution of the 8 filtered variables included in the final SEM analysis

Two-layer Variables	One-layer Variables	Original Variables	Detailed distribution
Overall index	Concomitant variables	Age at diagnosis	50.99 ± 36.38 months
		Abnormal pregnancy history	23.13% (+)
			76.87% (−)
	Structural indices	AL	21.75 ± 2.06 mm
		Area	55% (Extensive)
			45% (Limited)
		Density	37.5% (Dense)
			63.5% (Non-dense)
		Location	60.63% (Central)
			39.37% (Peripheral)
	Functional indices	IOP	15.12 ± 6.88 mmHg
		UCVA	0.77 ± 0.44 (logMAR)

A total of 160 participants from the CCPMOH database and 8 filtered variables were included in the final SEM analysis. The mean age of the included participants was 50.99 months ± 36.38 months, and 23.13% (*n* = 37) of our patients had an abnormal pregnancy history. For the STR network, the mean value of AL was 21.75 mm ± 2.06 mm; 55% (*n* = 88) of our patients had an extensive area; 37.5% (*n* = 60) of our patients had dense opacity, and 60.63% (*n* = 97) of our patients had opacity at central location. For the FUN network, the mean IOP value was 15.12 mmHg ±6.88 mmHg, and the mean UCVA value (logMAR) was 0.77 ± 0.44

*AL* Axial length, *IOP* Intraocular pressure, *UCVA* uncorrected visual acuity

SEM was conducted to statistically test the interrelationships of the constructs and their relationships with the structural, functional, and overall components in our study population. Prior to modeling the relationship between latent variables, a measurement model was evaluated for each dietary behavior component. This step involves a confirmatory factor analysis to reveal the relationship between the latent variables and their indicator variables. The following step (i.e., testing of the structural model) estimates the strength of the relationships between these latent variables. It also allows for an examination of the direct and indirect effects among the constructs in the model. The data were examined prior to modeling to ensure that they met the assumptions of the proposed SEM and were analyzed using the robust weighted least squares procedure in Mplus 7.0 (Mplus, Los Angeles, Calif) [21].

To evaluate the goodness of fit of the model, the χ^2^-value was calculated together with the degrees of freedom and four other indices as follows: the root mean square error of approximation (RMSEA), the Tucker-Lewis index (TLI), the comparative fit index (CFI), and the weighted residual root mean square residual (WRMR). Values below 0.08 for RMSEA, below 1.0 for WRMR and above 0.90 for TFI and CFI indicate an acceptable fit of the data to the hypothesized model (details in Additional file 1).

## Results

### Participant characteristics

A total of 160 participants from the CCPMOH database with 8 filtered variables were included in the final SEM analysis (details in Table 2). The mean age of the included participants was 50.99 months ± 36.38 months and 23.13% (*n* = 37) of our patients had an abnormal pregnancy history. For the STR network, the mean AL value was 21.75 mm ± 2.06 mm, 55% (*n* = 88) of our patients had an extensive area, 37.5% (*n* = 60) of our patients had dense opacity, and 60.63% (*n* = 97) of our patients’ opacities had central locations. For the FUN network, the mean IOP value was 15.12 ± 6.88 mmHg, and the mean UCVA value (logMAR) was 0.77 ± 0.44.

### Goodness-of-fit of the models

As shown in Table 3, the overall fit of the measurement and the structural models was satisfactory (χ^2^ = 26.093, *df* = 16, *P* = 0.1113; RMSEA = 0.053, *P* = 0.422; CFI = 0.995; TFI = 0.992; WRMR = 0.781). Finally, the estimates of the SEM constructs are presented in Table 4.

**Table 3:** Summary of statistics of the goodness-of-fit indices

Goodness-of-fit index	Results
χ^2^ (Chi-square value)	χ^2^ = 26.093, *df* = 16, *P* = 0.1113
RMSEA	0.053, *P* = 0.422
CFI	0.995
TFI	0.992
WRMR	0.781

The overall fit of the measurement and the structural models was satisfactory (*χ* ^*2*^ = 26.093, *df* = 16, *P* = 0.1113; RMSEA = 0.053, *P* = 0.422; CFI = 0.995; TFI = 0.992; WRMR = 0.781)

*RMSEA* Root mean square error of approximation, *TLI* Tucker- Lewis index, *CFI* Comparative fit index, *WRMR* Weighted residual root mean square residual

**Table 4:** Modeling estimation indices of the SEM constructs

Variables		Coefficient estimates value	Standardized coefficient estimation	Standard error	*P* value
Structural indices	AL	1.000	0.615	-	-
	Area	−0.203	−0.243	0.092	0.027
	Density	−0.543	−0.570	0.106	0.000
	Location	−0.308	−0.357	0.107	0.004
Functional indices	IOP	1.000	0.342	-	-
	UCVA	0.054	0.571	0.033	0.098
Overall index by	STRI	1.000	1.046	-	-
	FUNI	1.785	0.974	0.493	0.000
Overall index on	Age at diagnosis	0.027	0.753	0.004	0.000
	Abnormal pregnancy history	−0.787	−0.260	0.266	0.003

*AL* Axial length, *IOP* Intraocular pressure, *UCVA* uncorrected visual acuity, *STRI* Structural indices, *FUNI* Functional indices

### Interrelationships of the constructs

**Fig. 2:**
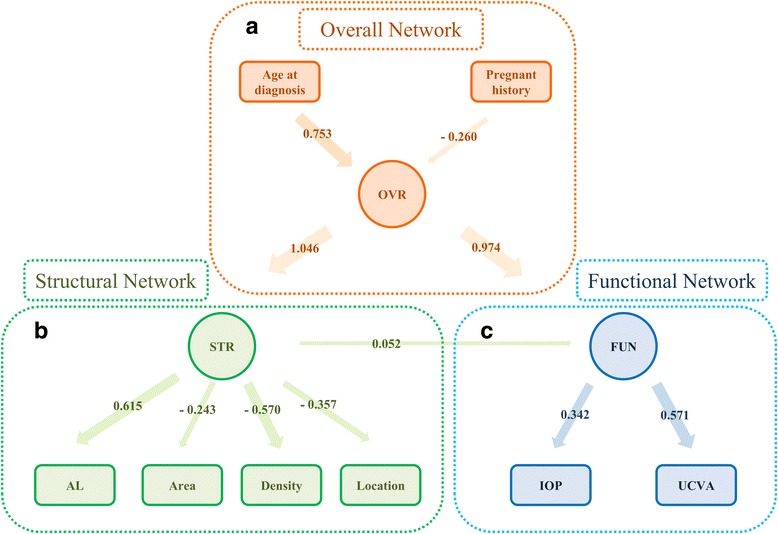
The standardized SEM architecture and interrelationships of the constructs. **a** The overall network exhibited a positive correlation with the FUN (0.974) and STR indices (1.046). The age at diagnosis (0.753) was positively correlated with the overall index, while an abnormal pregnancy experience (−0.26) exhibited a negative relationship with the overall index. **b** The lesion area (−0.243), density (−0.57) and location (−0.357) were all negatively correlated with the STR index. Meanwhile, the AL variable has the positive correlation with the STR index (0.615). **c**, Increased UCVA was directly associated with a higher FUN index (0.571), and the IOP value was positively correlated with the FUN index (0.342). *AL* Axial length, *IOP* Intraocular pressure, *UCVA* uncorrected visual acuity, *BCVA* best-corrected visual acuity, *STR* Structural indices, *FUN* Functional indices, *OVE* Overall indices

The standardized SEM architecture and interrelationships of the constructs are displayed in Fig. 2.

#### STR networks

The lesion area (−0.243), density (−0.57), and location (−0.357) simultaneously had negative correlations with the STR index. The AL variable had a positive correlation with the STR index (0.615).

#### FUN networks

The presence of higher UCVA was positively associated with a higher FUN index (0.571), suggesting that patients with higher UCVA will accordingly have better visual functions. Furthermore, the IOP value was positively correlated with the FUN index (0.342). Because the main range of the IOP is below 21 mmHg (normal range), this result mainly reveals the functional support role of IOP, which is necessary for visual maintenance.

#### Overall networks

A positive relationship in the model was the link among the FUN (0.974) and STR indices (1.046) and the overall network index, which indicates that function and structure are two of the main dimensions for overall disease evaluation, with structure forming the base of visual function (0.052). The age at diagnosis (0.753) was positively correlated with the overall index, whereas an abnormal pregnancy experience (−0.26) had a negative relationship with the overall index. These results demonstrate that age is an important indicator for the visual maturation and that an abnormal pregnancy experience will have a detrimental influence on overall visual function.

### Clinical interpretations

The results from SEM network quantifiably illuminate the effect of eight potential factors (four risk factors: area, density, location, and abnormal pregnancy experience; four beneficial factors: AL, UCVA, IOP value, and age at diagnosis). For structural effect, extensive lesion area, dense opacity and central location will present increasing severity of cataract but AL growth will be a positive indicator for the better structural maturity. Furthermore, both UCVA and IOP value in normal range presented beneficial functional effect and could be the positive evaluation indicators for patients. Additionally, patients with earlier diagnosis age and abnormal pregnancy experience will be the indicators for a more severe status. All these identified factors are also valuable references and indicators for patients’ prognoses.

## Discussion

### It is imperative to study rare and complex diseases in a systematic manner

Studying rare and complex disease is a great challenge for researchers and medical practitioners [22]. Rare and complex diseases tend to be caused by a system, with interconnected entities being modeled as nodes and their connections as edges to comprise an intricate pathogenic network [23]. Therefore, it is always difficult for researchers to explicitly elucidate the principle nosogenesis, individual predisposition and related risk factors.

With the advance and popularization of medical detecting and monitoring equipment, medical practitioners should address an increasing number of clinical indices. However, quite a few examinations or clinical indices seem to be unnecessary or to provide ambiguous information for the diagnosis and the treatment decision, which is contrary to the goals of precision medicine [24, 25]. Therefore, it is imperative to uncover the interrelationship and the effectiveness of these potential clinical indices and risk factors and to integrate these elements to construct a holistic network capable of providing insights into rare disease evidence-based prevention and treatment.

To obtain sufficient evidence and data for rare diseases, we established the CCPMOH program and built a rare disease data integration platform [11]. Medical history, multiple structural indices, and comprehensive functional metrics were collected for the primary investigation. After pre-modeling, a total of 8 filtered variables were selected and included in the final SEM modeling.

### Implications from filtered variables: abnormal pregnancy history may be the principle risk factor among medical history for pediatric cataracts

Rare diseases, including pediatric cataracts, are always associated with birth defects [26]. However, pediatric cataracts present great genetic heterogeneity, and a considerable proportion of pediatric cataracts should be considered in the context of interactions among the environment and heredity [27]. Among the 8 filtered variables, abnormal pregnancy history was included but family hereditary history and abnormal parturition history were excluded from the final network. In addition to the final network contribution, these results indicate that abnormal pregnancy history may be the principle risk factor for pediatric cataracts. Previous studies reported that pediatric cataracts had an association with rubella infection [28, 29], which was in agreement with our findings on the abnormal pregnancy history. Therefore, more attentions should be focused on patients with abnormal pregnancy history in screening and early diagnostics of pediatric cataract.

### Our results are concordant with conventional medical knowledge and validate the reliability of the SEM network

To systematically model our clinical indices, we classified our records into two separated latent variances (structural indices or function indices) for two-layer SEM construction. Two essential steps are needed for the SEM clinical interpretation. First, we should holistically evaluate the SEM network using existing evidence and the clinical consensus. Our results indicate that the functional and structural networks have positive support from the overall network and that the structural network has a positive supporting effect on the functional network. Additionally, the lesion area, density and location have a negative effect on the structural network, and UCVA is a positive indicator for the functional network. These results are concordant with conventional medical knowledge and validate the reliability of the SEM network.

### Implications from SEM network index: the risk or beneficial effects of the potential factors are confirmed to provide insights into rare disease prevention and treatment

After verifying the reliability of the model in the preliminary test, the second step is to explain the model and provide novel and valuable clinical information. For the structural network, AL presents a positive effect in our study population. The main study population was younger than 2 years of age; thus, in the early stage, increasing AL is a beneficial indictor for eyeball development instead of myopia progression. For the functional network, the IOP plays as a supporting role, which reveals that an IOP in the normal range (the main range of our IOP is below 21 mmHg) is not a risk factor for glaucoma but instead is a supporting factor for visual function and a positive nutrient media for the functional component. For the overall network, age at diagnosis is also a positive indicator, which demonstrates that the severe pediatric cataract is more likely to be diagnosed in the early stage. These implications could be an important supplement to the mainstream theory of pediatric cataract.

### Implications from weight of index: AL, density, UCVA and age at diagnosis served as the dominant factors and might be emphasized in our regular clinical practice

The weight of each index is also valuable information for the clinical interpretation. For the structural network, AL and density are the dominant factors and can be considered key indicators for severity and prognosis. For the functional network, UCVA plays the main role along with the subordinate beneficial effect from the IOP. The functional and structural networks almost equally provide a contribution to the overall network. Furthermore, the age at diagnosis is more crucial than an abnormal pregnancy experience in the overall network. Therefore, AL, density, UCVA and age at diagnosis might be emphasized in our regular clinical practice.

### Acceptable fitting of SEM network and study limitation

We performed the goodness-of-fit validation to ensure the meaning and reliability of the SEM network. The key indicators, χ^2^-values and RMSEA indicated an acceptable fit of the data to our model along with the TFI, CFI, and WRMR, which supported the same conclusion. Although the modeling reliability of our variables was not sufficiently ideal to show great explanation power, the overall fitting of the SEM network is acceptable.

The main limitation of our study is the data quality. Two main effects (missing data and inevitable errors) caused by data quality are introduced as follows. Missing data will directly influence the model fitting; thus, the reason for the exclusion of the primary indices should not be merely interpreted by their limited or subordinate effect on disease. Notably, some valuable indices might be excluded by the effect of the missing data. Moreover, the missing data could be a possible explanation for the unsatisfactory modeling reliability. Additionally, uncooperative measurement is common for our regular examination procedure because the majority of our patients are under 2 years of age, which causes fairly inevitable measuring errors. Furthermore, the VA evaluation is a somewhat subjective measurement and the medical history is a retrospective inquiry, which might present less accuracy. Our future study aims are to include a more dimensional and more complete rare disease dataset with a larger sample size for modeling and data mining.

## Conclusions

In conclusion, our study proposed a feasible pattern for data mining using a rare disease dataset. We integrated the multidimensional dataset and constructed a two-layer SEM network to identify valuable clinical indices and uncover the interrelationships and the effectiveness of these potential factors. Four risk factors and four beneficial factors were filtered and confirmed with reliable clinical implications. This research could promote rare disease research and prevention to substantially benefit rare disease patients.

## Additional file

Additional file 1: The detailed information for potential variables and evaluation indices are presented. (DOCX 16 kb)

## Abbreviations

AL: Axial length
BCVA: Best-corrected visual acuity
CCPMOH: Childhood cataract program of the chinese ministry of health
CFI: Comparative fit index
FUN: Functional index
ICD-10: International statistical classification of diseases and related health problems 10th revision
IOL: Intraocular lens
IOP: Intraocular pressure
OVE: Overall index
RMSEA: Root mean square error of approximation
SEM: Structural equation modeling
STR: Structural index
TLI: Tucker- lewis index
UCVA: Uncorrected visual acuity
VA: Visual acuity
WRMR: Weighted residual root mean square residual
