# Supplementary material for: Construction and implications of structural equation modeling network for pediatric cataract: a data mining research of rare diseases
Source: BMC Ophthalmol. 2017 May 19;17:74. doi: 10.1186/s12886-017-0468-5 (PMC5438536; doi:10.1186/s12886-017-0468-5)
Supplement: Additional file 1: — The detailed information for potential variables and evaluation indices are presented. (DOCX 16 kb) [file 12886_2017_468_MOESM1_ESM.docx]

**Supplementary Files**

**Detailed information for potential variables**

**Area, density, and location:** The area was defined as “extensive” when the opacity covered more than 50% of the pupil; otherwise, it was “limited”. The density was defined as “dense” when the opacity fully disrupted vision; otherwise, it was “non-dense”. The location was defined as “central” when the opacity fully covered the visual axis area; otherwise, it was “peripheral”.

**The intraocular pressure:** Each eye was measured 6 times by two different pediatric ophthalmologists to rule out operational errors and to ensure data reliability. Older and cooperative children, who could better tolerate a Goldman tonometry or pneumotonometry were simultaneously applied these tests to confirm the Tono-Pen results. The mean values of the measurements were used for the final analysis.

**Axial length measurement:** The A scan unit was equipped with a 10 MHz transducer probe, and the velocities were set as follows: 1,641 m/s for the cornea and lens and 1,532 m/s for the aqueous and vitreous humor. An applanation ultrasound was performed after the instillation of one drop of topical anesthetic (0.5% Alcaine, Alcon, USA) to the lower conjunctiva. Each eye was measured 10 times, and the mean measurements were used for the final analysis.

**Detailed information for evaluation indices**

The χ²-value has traditionally been used to test the hypothesis that the relationships suggested in the model provide a plausible explanation for the data (i.e., how well the proposed model structure fits the structure in the observed data set). However, this value is sensitive to sample size, with a large sample size increasing the power to reject the models. Therefore, other fit indices have been proposed to compensate for this problem. The root mean square error of approximation (RMSEA) is a measure of discrepancy between the true population model and the hypothesized model with unknown but optimally chosen parameter estimates. In other words, RMSEA favors a more parsimonious model with fewer parameters and is relatively insensitive to sample size because it is a population-based index. The comparative fit index (CFI), the Tucker-Lewis index (TLI), and the weighted residual root mean square residual (WRMR) compare the fit of the hypothesized model to that of a baseline or null model, where all parameters are assumed to be independent.
